# The STING1-MYD88 complex drives ACOD1/IRG1 expression and function in lethal innate immunity

**DOI:** 10.1016/j.isci.2022.104561

**Published:** 2022-06-08

**Authors:** Feng Chen, Runliu Wu, Jiao Liu, Rui Kang, Jinbao Li, Daolin Tang

**Affiliations:** 1 Department of Anesthesiology, Shanghai General Hospital, Shanghai Jiao Tong University School of Medicine, Shanghai 200080, China; 2 Department of Surgery, UT Southwestern Medical Center, Dallas, TX 75390, USA; 3 DAMP Laboratory, Guangzhou Municipal and Guangdong Provincial Key Laboratory of Protein Modification and Degradation, The Third Affiliated Hospital, Guangzhou Medical University, Guangzhou, Guangdong 510510, China

**Keywords:** Biological sciences, Immunology, Immune response

## Abstract

ACOD1 (also known as IRG1) has emerged as a regulator of immunometabolism that operates by producing metabolite itaconate. Here, we report a key role of STING1 (also known as STING and TMEM173) in mediating ACOD1 expression in myeloid cells in response to toll-like receptor (TLR) signaling. The activation of STING1 through exogenous cyclic dinucleotides (e.g., 3′3′-cGAMP) or endogenous gain-of-function mutation (e.g., V155M) enhances lipopolysaccharide-induced ACOD1 expression and itaconate production in macrophages and monocytes, whereas the deletion of STING1 blocks this process. The adaptor protein MYD88, instead of DNA sensor cyclic GMP-AMP synthase (CGAS), favors STING1-dependent ACOD1 expression. Mechanistically, MYD88 directly blocks autophagic degradation of STING1 and causes subsequent IRF3/JUN-mediated ACOD1 gene transcription. Consequently, the conditional deletion of STING1 in myeloid cells fails to produce ACOD1 and itaconate, thereby protecting mice against endotoxemia and polymicrobial sepsis. Our results, therefore, establish a direct link between TLR4 signaling and ACOD1 expression through the STING1-MYD88 complex during septic shock.

## Highlights

• The MYD88-STING1 protein complex is required for lipopolysaccharide (LPS)-induced ACOD1 expression
• The MYD88-STING1 protein complex prevents autophagic degradation of STING1
• The IRF3-JUN transcription factor complex favors ACOD1 upregulation
• STING1-mediated itaconate production promotes experimental sepsis

## Graphical abstract

**Figure:**
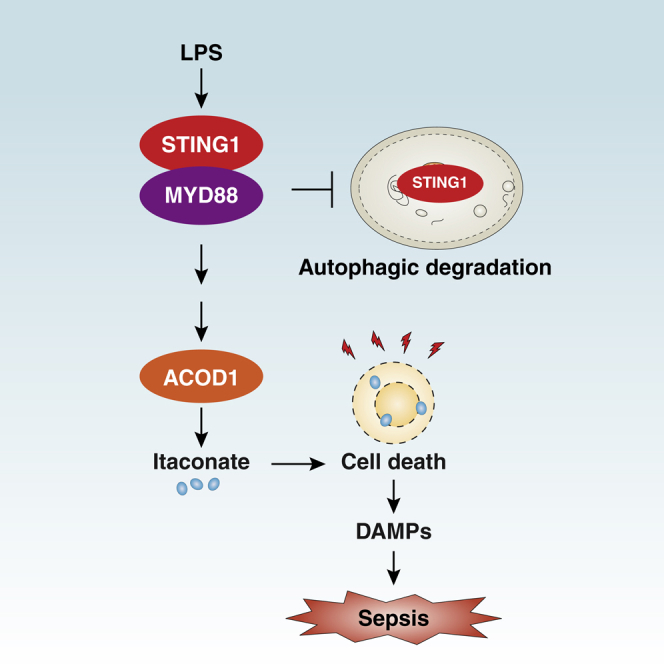

## Introduction

Sepsis is one of the oldest and most elusive syndromes in medicine that is defined by a dysregulated host response to pathogen infection (Singer et al., 2016). Monocytes and macrophages are the main sources of the production of immune mediators in septic shock, which can be activated by pathogen-associated molecular patterns (PAMPs) using a set of receptors called pattern recognition receptors (Wiersinga et al., 2014). Several metabolic processes in immune cells, including aerobic glycolysis, the tricarboxylic acid cycle, fatty acid metabolism, and itaconate metabolism, promote the activation or quiescence of the inflammatory response (Jung et al., 2019; O'Neill et al., 2016; Wu et al., 2022). Understanding the process, modulation, and function of immunometabolism is critical for the development of therapies for sepsis (Koutroulis et al., 2019).

Lipopolysaccharide (LPS) is a component and PAMP of the outer membrane of the Gram-negative bacteria that plays a strong role in host-pathogen interaction by activating toll-like receptor 4 (TLR4) on plasma membranes (Poltorak et al., 1998) or caspase 11 in the cytoplasm (Shi et al., 2014). The activation of the LPS-TLR4 pathway controls the expression of multiple genes involved in innate immunity (Guha and Mackman, 2001). In addition to cytokines and chemokines, the mitochondrial enzyme aconitate decarboxylase 1 (ACOD1, also known as IRG1), was identified as a highly LPS-induced gene in macrophages (Lee et al., 1995). The biological effects of ACOD1 mainly depend on the generation of endogenous itaconate with anti-inflammatory activity (Bambouskova et al., 2018, 2021; Cordes et al., 2016; Hooftman et al., 2020; Lampropoulou et al., 2016; Nair et al., 2018; Swain et al., 2020). Itaconate also inhibits the function of phagocytes to eliminate pathogens (Michelucci et al., 2013; Naujoks et al., 2016), indicating a dual role of the ACOD1-itaconate axis in infection (Luan and Medzhitov, 2016; Wu et al., 2020).

Stimulator of interferon response CGAMP interactor 1 (STING1, also known as TMEM173 or STING) is a well-known regulator of the cyclic GMP-AMP synthase (CGAS)-dependent DNA sensor pathway, which drives antiviral immunity by producing type I interferons (IFNs) (Ishikawa and Barber, 2008; Ishikawa et al., 2009; Sun et al., 2009; Zhong et al., 2008). Increasing evidence highlights that a dysfunctional STING1 pathway is implicated in sterile inflammation and infection (Barber, 2015; Motwani et al., 2019; Zhang et al., 2022a). We and others have recently demonstrated that excessive activation of the STING1 pathway contributes to cytokine storms, systemic coagulation, and multiple organ failure in experimental models of sepsis (Ge et al., 2019; Heipertz et al., 2017; Hu et al., 2019; Kang et al., 2017; Ning et al., 2020; Wu et al., 2020). However, whether STING1 affects ACOD1-related immunometabolism remains obscure.

In this study, we provide the first evidence that STING1 mediates LPS-induced ACOD1 expression by binding to adaptor protein myeloid differentiation marker 88 (MYD88), rather than through a CGAS-dependent signaling pathway. The deletion of STING1 in macrophages and monocytes limits LPS-induced ACOD1 expression as well as itaconate production, thereby preventing septic death in mice. These findings establish a framework for understanding the interaction of STING1 and TLR signals in the control of immunometabolism.

## Results

### STING1 is required for lipopolysaccharide-induced ACOD1 expression

**Figure 1:**
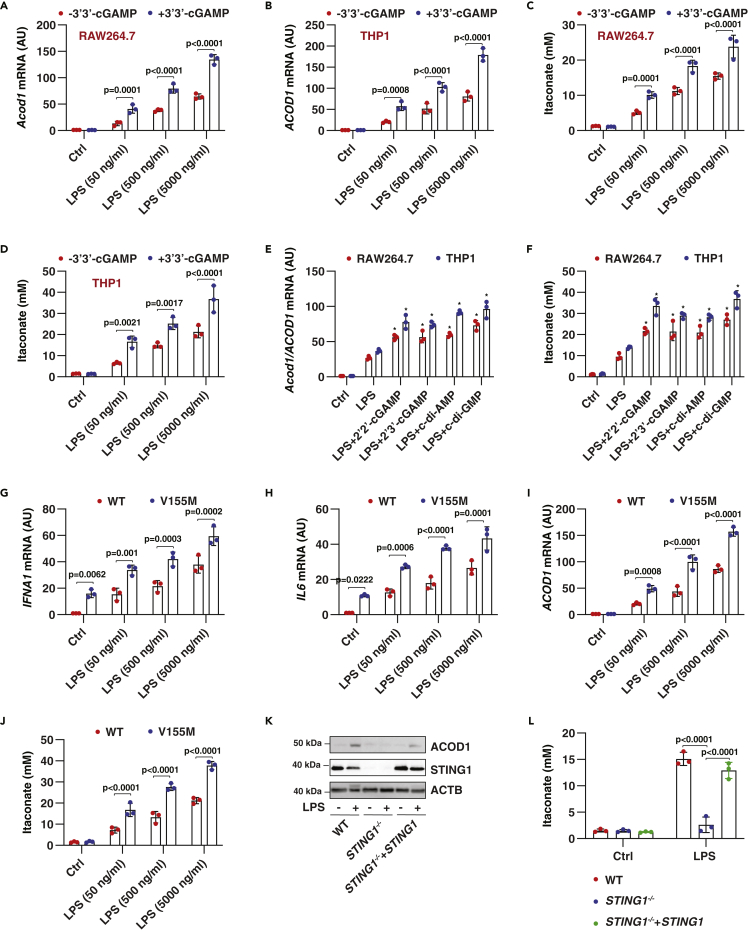
STING1 is required for LPS-induced ACOD1 expression (A–D) RAW264.7 and THP1 cells were treated with LPS (50-5000 ng/mL) in the absence or presence of 3′3′-cGAMP (10 μg/mL) for 6 h, and then *Acod1/ACOD1* mRNA and intracellular itaconate concentration were assayed (Data are presented as mean ± SD; n = 3 biologically independent samples; two-way ANOVA with Tukey’s multiple comparisons test). (E and F) RAW264.7 and THP1 cells were treated with LPS (500 ng/mL) in the absence or presence of 2′2′-cGAMP, 2′3′-cGAMP, c-di-AMP, or c-di-GMP at 10 μg/mL for 6 h, and then *Acod1/ACOD1* mRNA and intracellular itaconate concentration were assayed (Data are presented as mean ± SD; n = 3 biologically independent samples; p < 0.01 versus LPS along group; two-way ANOVA with Tukey’s multiple comparisons test). (G–J) WT and V155M-THP1 cells were treated with indicated LPS for 6 h, and then *IFNA1* mRNA, *IL6* mRNA, *ACOD1* mRNA, and intracellular itaconate concentration were assayed (Data are presented as mean ± SD; n = 3 biologically independent samples; two-way ANOVA with Tukey’s multiple comparisons test). (K) Western blot analysis of protein expression in indicated THP1 cells following treatment with LPS (500 ng/mL) for 6 h. (L) In parallel, intracellular itaconate concentration was assayed (Data are presented as mean ± SD; n = 3 biologically independent samples; two-way ANOVA with Tukey’s multiple comparisons test).

The *Acod1* gene was first cloned in RAW264.7 cells (a mouse macrophage-like cell line) following stimulation with 5000 ng/mL LPS for 1.5 h (Lee et al., 1995). Subsequent studies showed that the peak of ACOD1 expression in macrophages induced by LPS appears at 6 h (Li et al., 2013b). To determine the effects of STING1 on ACOD1 expression, we treated RAW264.7 cells with 50-5000 ng/mL LPS in the absence or presence of 3′3′-cGAMP for 6 h. The 3′3′-cGAMP is a cyclic dinucleotide (CDN) produced by bacteria and acts as a canonical STING1 ligand (Zhang et al., 2013). A qPCR analysis revealed that 3′3′-cGAMP alone cannot trigger *Acod1* mRNA expression, but significantly increases LPS-induced *Acod1* mRNA expression (Figure 1A). This enhancement of LPS-induced *ACOD1* gene expression by 3′3′-cGAMP was further confirmed in human monocyte cell line THP1 (Figure 1B). Accordingly, LPS-induced production of intracellular itaconate was enhanced in RAW264.7 and THP1 cells by 3′3′-cGAMP (Figures 1C and 1D). In addition to 3′3′-cGAMP, other naturally occurring CDNs (2′3′-cGAMP, c-di-AMP, and c-di-GMP) or synthetic STING1 ligand (2′2′-cGAMP) also increased LPS-induced *Acod1/ACOD1* gene expression (Figure 1E) and itaconate production (Figure 1F) in RAW264.7 and THP1 cells, highlighting a broad role of STING1 ligands in enhancing LPS-induced ACOD1 expression in myeloid cells.

Next, we examined whether the constitutive activation of STING1 enhances LPS-induced ACOD1 upregulation. We focused on V155M, which is a gain-of-function mutation that leads to the constitutive activation of STING1 and subsequent immune-mediated inflammatory disease in humans (Jeremiah et al., 2014; Liu et al., 2014). Compared to wild-type cells, V155M-THP1 cells had an increased basic expression of interferon-alpha 1 (*IFNA1*, best known as IFNα) (Figure 1G) and NF-κB target gene interleukin 6 (*IL6*) (Figure 1H), rather than the basic mRNA expression of *ACOD1* (Figure 1I). However, V155M-THP1 cells became more sensitive to LPS-induced ACOD1 expression (Figure 1I) and itaconate production (Figure 1J). Importantly, LPS-induced ACOD1 protein expression and itaconate production were blocked in *STING1*^*−/−*^ THP1 cells and this phenotype was rescued by the re-expression of STING1 (Figures 1K and 1L). Collectively, these findings strongly support the conclusion that STING1 plays a key role in regulating ACOD1 expression in LPS-activated monocytes and macrophages.

### MYD88, but not cyclic GMP-AMP synthase, mediates lipopolysaccharide-induced ACOD1 expression

**Figure 2:**
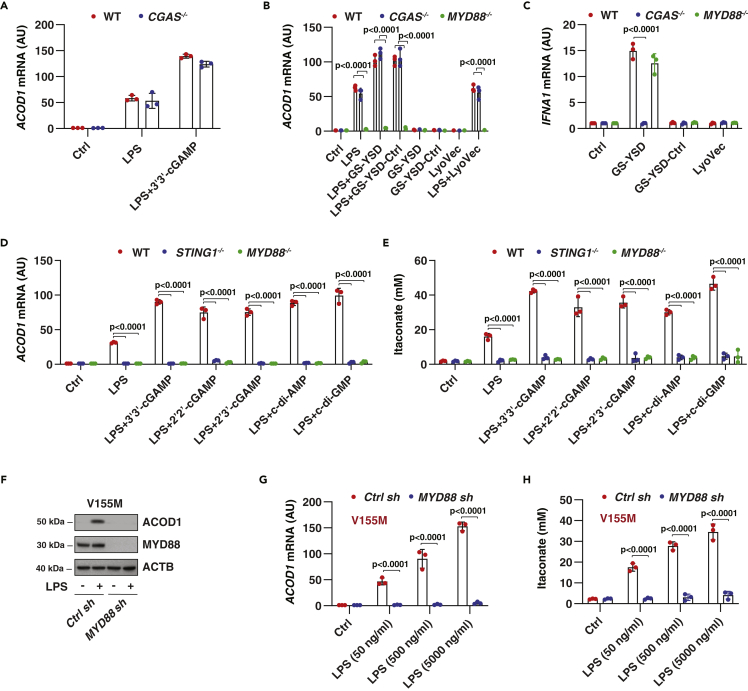
MYD88, but not CGAS, mediates LPS-induced ACOD1 expression (A–C) Indicated THP1 cells were treated with LPS (500 ng/mL) in the absence or presence of 3′3′-cGAMP (10 μg/mL), G3-YSD (1 μg/mL), G3-YSD-Ctrl (1 μg/mL), or LyoVec for 6 h, and then *ACOD1* or *IFNA1* mRNA was assayed (Data are presented as mean ± SD; n = 3 biologically independent samples; two-way ANOVA with Tukey’s multiple comparisons test). (D and E) Indicated THP1 cells were treated with LPS (500 ng/mL) in the absence or presence of 3′3′-cGAMP, 2′2′-cGAMP, 2′3′-cGAMP, c-di-AMP, or c-di-GMP at 10 μg/mL for 6 h, and then *ACOD1* mRNA and intracellular itaconate concentration were assayed (Data are presented as mean ± SD; n = 3 biologically independent samples; two-way ANOVA with Tukey’s multiple comparisons test). (F) Western blot analysis of protein expression in indicated V155M-THP1 cells following treatment with LPS (500 ng/mL) for 6 h. (G and H) Analysis of *ACOD1* mRNA and intracellular itaconate concentration in indicated V155M-THP1 cells following treatment with LPS (50-5000 ng/mL) for 6 h (Data are presented as mean ± SD; n = 3 biologically independent samples; two-way ANOVA with Tukey’s multiple comparisons test).

The cytosolic DNA sensor CGAS recognizes microbial or host DNA to catalyze the synthesis of cGAMP, thereby promoting the dimerization and activation of STING1 (Li et al., 2013a). However, the deletion of CGAS failed to block LPS-induced *ACOD1* mRNA expression in *CGAS*^*−/−*^ THP1 cells in the absence or presence of 3′3′-cGAMP (Figure 2A). G3-YSD is a 26-mer DNA sequence derived from the HIV-1 RNA genome (Herzner et al., 2015). Although G3-YSD is flanked with guanosine trimers (G3) that confer its CGAS agonist activity, G3-YSD control is flanked with cytidine trimers that abrogate CGAS activation (Herzner et al., 2015). Both G3-YSD and G3-YSD-Ctrl (but not cationic lipid-based transfection reagent LyoVec) enhanced LPS-induced ACOD1 expression in wild-type and *CGAS*^*−/−*^ THP1 cells (Figure 2B). G3-YSD and G3-YSD-control alone failed to induce ACOD1 expression (Figure 2B). As a positive control, the effect of G3-YSD (but not G3-YSD control) on the upregulation of *IFNA1* mRNA was blocked in *CGAS*^*−/−*^ cells (Figure 2C). Thus, CGAS is likely dispensable for STING1-mediated ACOD1 upregulation.

MYD88 is an adaptor protein for inflammatory signaling pathways downstream of multiple types of TLRs (Deguine and Barton, 2014). We, therefore, examined whether MYD88 is involved in the regulation of STING1-dependent ACOD1 expression. Unlike *CGAS*^*−/−*^ cells, the enhancement of LPS-induced ACOD1 expression by G3-YSD or G3-YSD-Ctrl was inhibited in *MYD88*^*−/−*^ cells (Figure 2B). However, the induction of IFNA1 by GS-YSD required CGAS, but not MYD88 (Figure 2C). Moreover, as in the response of *STING1*^−/−^ cells, in the absence or presence of STING1 ligands (3′3'-cGAMP, 2′2′-cGAMP, 2′3′-cGAMP, c-di-AMP, or c-di-GMP), *MYD88*^*−/−*^ THP1 cells lose the ability to produce ACOD1 and itaconate in response to LPS stimulation (Figures 2D and 2E). The suppression of MYD88 expression by shRNA in V155M-THP1 cells also blocked LPS-induced ACOD1 upregulation and itaconate production (Figures 2F-2H). Together, these data confirm the role of MYD88 in promoting STING1-mediated ACOD1 expression and function.

### The MYD88-STING1 protein complex prevents the autophagic degradation of STING1

**Figure 3:**
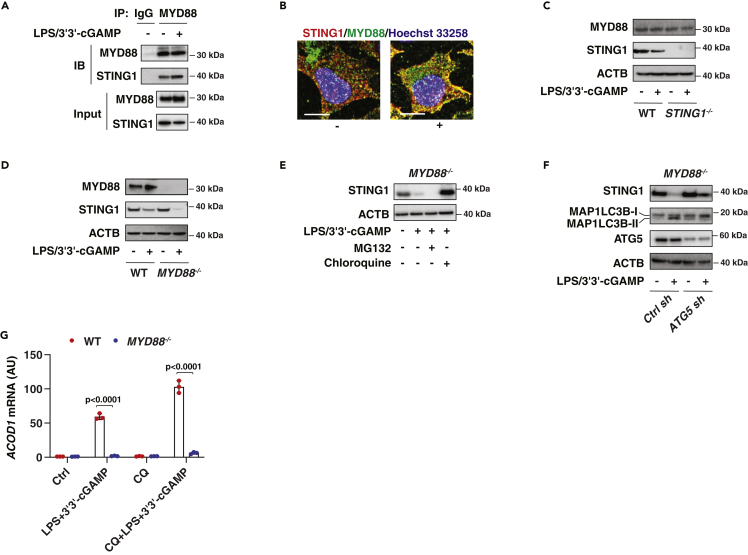
The MYD88-STING1 protein complex prevents autophagic degradation of STING1 (A) IP analysis of the MYD88-STING1 protein complex in THP1 cells following treatment with LPS (500 ng/mL) and 3′3′-cGAMP (10 μg/mL) for 6 h. (B) Representative colocalization images of MYD88 and STING1 in RAW264.7 cells in the presence or absence of LPS (500 ng/mL) and 3′3'-cGAMP (10 μg/mL) for 6 h. Bar = 10 μm. (C–F) Western blot analysis of protein expression in indicated THP1 cells following treatment with LPS (500 ng/mL) and 3′3′-cGAMP (10 μg/mL) in the absence or presence of chloroquine (50 μM) or MG132 (5 μM) for 6 h. (G) qPCR analysis of ACOD1 mRNA expression in indicated THP1 cells following treatment with LPS (500 ng/mL) and 3′3′-cGAMP (10 μg/mL) in the absence or presence of chloroquine (CQ; 50 μM) for 6 h (Data are presented as mean ± SD; n = 3 biologically independent samples; two-way ANOVA with Tukey’s multiple comparisons test).

As both MYD88 and STING1 are adaptor proteins in innate immunity, we hypothesized that STING1 and MYD88 may form a protein complex to regulate signal transduction. Indeed, immunoprecipitation analysis revealed that the STING1-MYD88 protein complex was present in THP1 cells, and this complex was mildly increased by stimulation with LPS/3′3′-cGAMP (Figure 3A). Image analysis confirmed the colocalization between STING1 and MYD88 in RAW264.7 cells (Figure 3B). However, the deletion of *MYD88* increased LPS/3′3′-cGAMP-induced STING1 protein degradation in *MYD88*^*−/−*^ cells, whereas the depletion of *STING1* had no effects on the level of MYD88 protein in *STING1*^*−/−*^ cells (Figures 3C and 3D). Thus, the STING1-MYD88 complex prevents STING1 protein degradation in activated THP1 cells.

To further define the mechanism of STING1 protein degradation in *MYD88*^*−/−*^ cells, we treated *MYD88*^*−/−*^ cells with chloroquine (an autophagy inhibitor) or MG132 (a proteasome inhibitor). Chloroquine, instead of MG132, blocked LPS/3′3′-cGAMP-induced STING1 degradation in *MYD88*^*−/−*^ cells (Figure 3E). The hypothesis that autophagy mediates STING1 protein degradation was further confirmed in *MYD88*^*−/−*^ cells after the knockdown of autophagy-related 5 (ATG5) (Figure 3F), a key driver of autophagosome formation. Accordingly, LPS/3′3′-cGAMP-induced production of microtubule-associated protein one light chain three beta (MAP1LC3B)-II, a marker of autophagosomes (Klionsky et al., 2021), was inhibited by the knockdown of *ATG5* (Figure 3F). Although chloroquine increased STING1 expression in activated *MYD88*^*−/−*^ cells, chloroquine failed to restore LPS/3′3′-cGAMP-induced ACOD1 expression in *MYD88*^*−/−*^ cells (Figure 3G). In contrast, the inhibition of autophagy by chloroquine increased LPS/3′3′-cGAMP-induced ACOD1 upregulation in wild-type THP1 cells (Figure 3G). These findings further confirm that the lack of STING1 or MYD88 leads to the suppression of inducible ACOD1 expression.

### STING1 and MYD88 selectively mediate toll-like receptor signaling to induce ACOD1 expression

**Figure 4:**
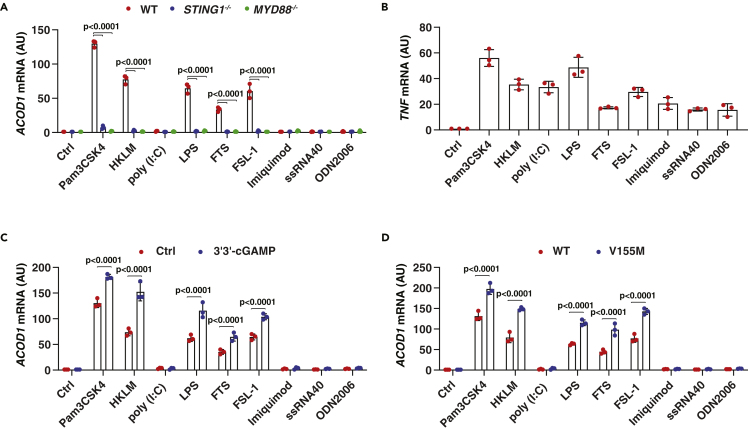
STING1 and MYD88 selectively mediate TLR signaling to induce ACOD1 expression (A) Indicated THP1 cells were stimulated with pam3CSK4 (1 ng/mL), HKLM (10^7^ cells/mL), poly (I:C) (10 μg/mL), LPS (500 ng/mL), FLA-ST (100 ng/mL), FSL1 (0.1 ng/mL), imiquimod (5 μg/mL), ssRNA40 (5 μg/mL), or ODN2006 (10 μg/mL) for 6 h and the mRNA level of ACOD1 was assessed (Data are presented as mean ± SD; n = 3 biologically independent samples; two-way ANOVA with Tukey’s multiple comparisons test). (B) Wild-type THP1 cells were stimulated with indicated TLR ligands for 3 h and the mRNA level of TNF was assessed (Data are presented as mean ± SD; n = 3 biologically independent samples). (C) Wild-type THP1 cells were stimulated with indicated TLR ligands in the absence or presence of 3′3′-cGAMP (10 μg/mL) for 6 h and the mRNA level of ACOD1 was assessed (Data are presented as mean ± SD; n = 3 biologically independent samples; two-way ANOVA with Tukey’s multiple comparisons test). (D) Wild-type and V115M THP1 cells were stimulated with indicated TLR ligands for 6 h and the mRNA level of ACOD1 was assessed (Data are presented as mean ± SD; n = 3 biologically independent samples; two-way ANOVA with Tukey’s multiple comparisons test). The TLR ligand concentration used in panels B-D is the same as for panel A.

The TLR family contains different members and their activation can be divided into MYD88-dependent (e.g., TLR1, TLR2, TLR4, TLR5, TLR6, TLR7, TLR8, and TLR9) and -independent (e.g., TLR3) pathways (Deguine and Barton, 2014). We next asked whether the STING1-MYD88 protein complex also contributes to inducible ACOD1 expression in response to other TLR ligands. THP1 and RAW264.7 cells were stimulated with classical TLR ligands, including Pam3CSK4 (a ligand for TLR1/2), HKLM (TLR2), poly (I:C) (TLR3), LPS (TLR4), FTS (TLR5), FSL-1 (TLR6), imiquimod (TLR7), ssRNA40 (TLR8), and ODN2006 (TLR9). Although many TLR ligands have been reported to induce ACOD1 expression under different conditions (Wu et al., 2020), we found that only Pam3CSK4 (TLR1/2), HKLM (TLR2), LPS (TLR4), FTS (TLR5), or FSL-1 (TLR6) had activity to trigger ACOD1 expression in THP1 cells (Figure 4A). Poly (I:C) (TLR3), imiquimod (TLR7), ssRNA40 (TLR8), and ODN2006 (TLR9) did not induce ACOD1 expression in THP1 cells (Figure 4A). As a positive control, these TLR ligands induced mRNA expression of the classical pro-inflammatory cytokine tumor necrosis factor (TNF) in THP1 cells (Figure 4B).

Notably, compared with other TLR ligands, the synthetic bacterial triacylated lipopeptide Pam3CSK4 showed the strongest activity in inducing ACOD1 expression (Figure 4A). The deletion of MYD88 or STING1 blocked Pam3CSK4 (TLR1/2), HKLM (TLR2), LPS (TLR4), FTS (TLR5), or FSL-1 (TLR6)-induced ACOD1 upregulation (Figure 4A). In contrast, treatment with 3′3′-cGAMP or a gain-of-function mutation of STING1 (V155M) increased inducible expression of ACOD1 in THP1 cells following stimulation with Pam3CSK4, HKLM, LPS, FTS, or FSL-1, which did not occur during stimulation by poly (I:C), imiquimod, ssRNA40, or ODN2006 (Figures 4C and 4D). Based on these analyses, we concluded that the activation of the STING1 pathway sensitizes LPS-induced ACOD1 expression.

### The IRF3-JUN transcription factor complex favors ACOD1 upregulation

**Figure 5:**
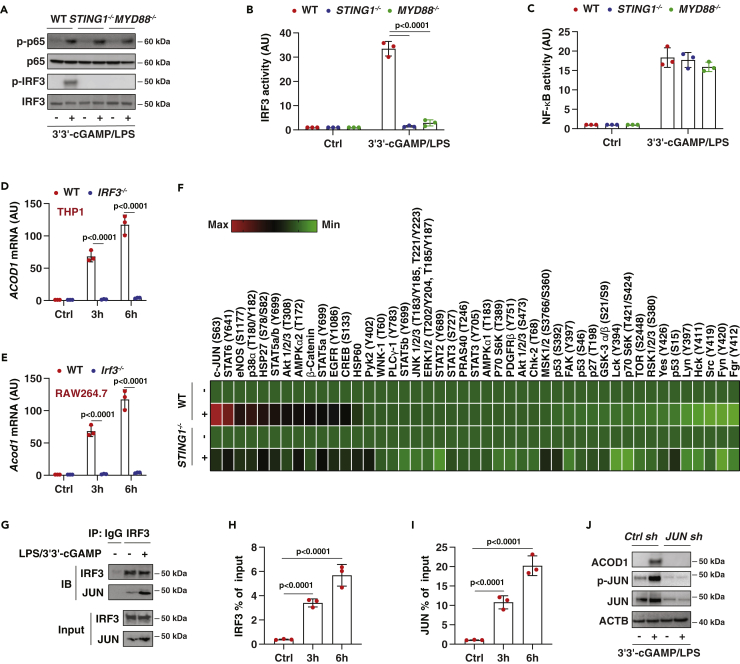
The IRF3-JUN transcription factor complex is required for ACOD1 upregulation (A) Western blot analysis of p-p65 and *p*-IRF3 in indicated THP1 cells following stimulation with 3′3'-cGAMP/LPS for 6 h. (B and C) In parallel, the activity of IRF or NF-κB was assayed by luciferase reporter gene assay (Data are presented as mean ± SD; n = 3 biologically independent samples; two-way ANOVA with Tukey’s multiple comparisons test). (D and E) Analysis of ACOD1 mRNA in indicated THP1 or RAW264.7 cells following stimulation with 3′3′-cGAMP/LPS for 3 and 6 h (Data are presented as mean ± SD; n = 3 biologically independent samples; two-way ANOVA with Tukey’s multiple comparisons test). (F) Heatmap of kinase phosphorylation changes in indicated THP1 cells following 3′3'-cGAMP/LPS stimulation for 6 h. (G) IP analysis of IRF3-JUN protein complex in THP1 cells following stimulation with 3′3′-cGAMP/LPS for 6 h. (H and I) ChIP-qPCR analysis of the binding of IRF3 and JUN on the promoter of ACOD1 in THP1 cells following stimulation with 3′3′-cGAMP/LPS for 3 and 6 h (Data are presented as mean ± SD; n = 3 biologically independent samples; *t* test). (J) Western blot analysis of ACOD1 in indicated THP1 cells following stimulation with 3′3′-cGAMP/LPS for 6 h.

We next sought to identify the transcription factor responsible for STING1-dependent ACOD1 upregulation. We focused on interferon regulatory factor 3 (IRF3) and nuclear factor-kappa B (NF-κB), two well-known transcription factors that regulate STING1-dependent cytokine expression (Barber, 2015; Motwani et al., 2019). Western blot analysis revealed that the phosphorylation of NF-κB p65 was not affected by the depletion of STING1 or MYD88 in THP1 cells in response to 3′3′-cGAMP/LPS (Figure 5A). In contrast, 3′3′-cGAMP/LPS-induced phosphorylation of IRF3 was diminished in *STING1*^*−/−*^ or *MYD88*^*−/−*^ cells (Figure 5A). Transcription factor activity assays further confirmed the effect of STING1 and MYD88 in mediating IRF3 activation (Figure 5B), rather than NF-κB activation (Figure 5C), in THP1 cells following stimulation with 3′3′-cGAMP/LPS.

To define the direct role of IRF3 in regulating ACOD1 expression, we used IRF3-deficient cells. The depletion of IRF3 in THP1 (Figure 5D) and RAW264.7 (Figure 5E) cells inhibited 3′3′-cGAMP/LPS-induced ACOD1 mRNA expression. These results support a significant role for IRF3 in mediating inducible ACOD1 expression in macrophages and monocytes.

It is worth noting that 3′3′-cGAMP alone increased IRF3 activation (Zhang et al., 2013), but did not trigger ACOD1 expression (Figure 1A), indicating that IRF3-mediated ACOD1 expression requires other co-regulators. This possibility was examined using a human phospho-kinase antibody array. We identified that the phosphorylation of transcription factor JUN at Ser63 was upregulated by 3′3′-cGAMP/LPS and this process was impaired in *STING1*^*−/−*^ cells (Figure 5F). Immunoprecipitation analysis revealed an increase in the IRF3-JUN protein complex formation in response to 3′3′-cGAMP/LPS (Figure 5G). As JUN is an LPS-inducible gene, the total JUN expression was also upregulated by 3′3′-cGAMP/LPS (Figure 5G). Chromatin immunoprecipitation (ChIP) analysis further showed that IRF3 and JUN bind directly to the promoter of ACOD1 in activated THP1 cells (Figures 5H and 5I). Functionally, the suppression of *JUN* expression by shRNA blocked 3′3′-cGAMP/LPS-induced ACOD1 expression in THP1 cells (Figure 5J). Altogether, these findings identify the activation of the IRF3-JUN transcription factor complex favors STING1-dependent ACOD1 expression.

### STING1-mediated itaconate production promotes experimental sepsis

**Figure 6:**
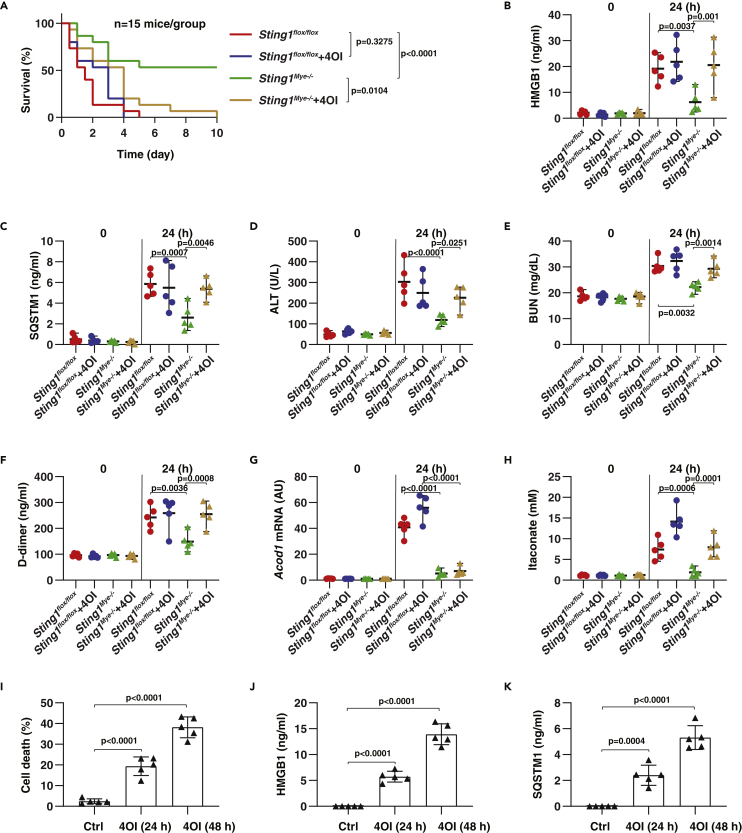
STING1-mediated itaconate production promotes endotoxemia (A) Analysis of animal survival in indicated mice with or without repeated intraperitoneal administration of 4OI (50 mg/kg) at 2, 24, 48, and 72 h after LPS (15 mg/kg) treatment (n = 15 mice/group; Kaplan-Meier survival analysis). (B–H) In parallel, indicated plasma biomarkers (C-F), as well as *Acod1* mRNA (G) and itaconate (H) in peritoneal macrophages, were assayed (Data are presented as mean ± SD; n = 5 mice/group; one-way ANOVA with Tukey’s multiple comparisons test). (I–K) Peritoneal macrophages were treated with 4OI (1 mM) for 24 and 48 h, and cell death and the release of HMGB1 and SQSTM1 were assayed (Data are presented as mean ± SD; n = 5 biologically independent samples; one-way ANOVA with Tukey’s multiple comparisons test).

**Figure 7:**
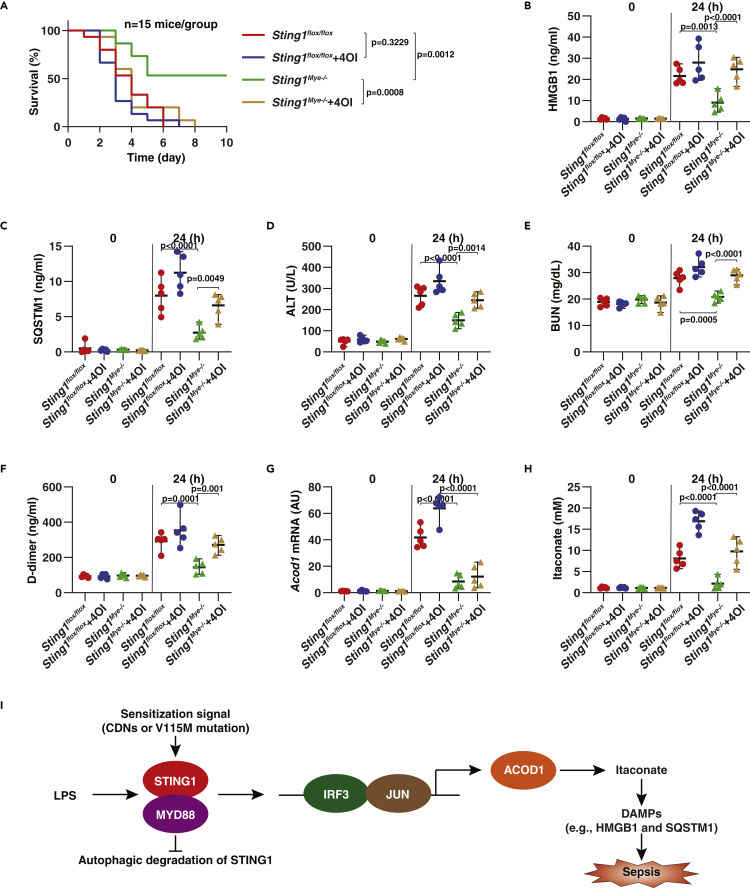
STING1-mediated itaconate production promotes polymicrobial sepsis (A) Analysis of animal survival in indicated mice with or without repeated intraperitoneal administration of 4OI (50 mg/kg) at 2, 24, 48, and 72 h following CLP procedure (n = 15 mice/group; Kaplan-Meier survival analysis). (B–H) In parallel, indicated plasma biomarkers (C-F), as well as *Acod1* mRNA (G) and itaconate (H) in peritoneal macrophages, were assayed (Data are presented as mean ± SD; n = 5 mice/group; one-way ANOVA with Tukey’s multiple comparisons test). (I) Schematic summary of the role of STING1 in driving ACOD1 expression and itaconate production for sepsis.

To determine the significance of STING1-mediated ACOD1 expression *in vivo*, we used two mouse models, including one for endotoxemia as well as polymicrobial sepsis induced by cecum ligation and puncture (CLP). Consistent with our previous studies (Zhang et al., 2020), the conditional deletion of STING1 in myeloid cells (termed *Sting1*^*Mye−/−*^ mice) prevented the animal death caused by endotoxemia (Figure 6A) or CLP (Figure 7A), and the production of circulating damage-associated molecular patterns (DAMPs; e.g., high-mobility group box 1 [HMGB1] (Wang et al., 1999) and sequestosome 1 [SQSTM1] (Zhou et al., 2020)), tissue-dysfunction markers (e.g., alanine aminotransferase [ALT] and blood urea nitrogen [BUN]), and blood clotting marker D-dimer (Figures 6C-6F and 7C-7F), supporting that STING1 is a mediator of septic shock. Subsequent analysis of isolated peritoneal macrophages confirmed that STING1 is required for inducible ACOD1 expression and itaconate production in the setting of experimental endotoxemia or polymicrobial sepsis (Figures 6G, 6H, 7G, and 7H).

We next evaluated the impact of itaconate on an antiseptic phenotype of *Sting1*^*Mye−/−*^ mice. We administered 4-octyl itaconate (4OI), the cellular permeable derivate of itaconate, to *Sting1*^*flox/flox*^ (control group) and *Sting1*^*Mye−/−*^ mice. Unlike previous studies that showed that 50 mg/kg of 4OI can protect against endotoxemia (Mills et al., 2018), we did not observe any statistical difference in 4OI on animal deaths in control mice during endotoxemia or CLP-induced sepsis (Figures 6A and 7A). However, the protection against septic death experienced by *Sting1*^*Mye−/−*^ mice was reversed by the administration of itaconate (Figures 6A and 7A). Accordingly, plasma HMGB1, SQSTM1, ALT, and BUN in septic *Sting1*^*Mye−/−*^ mice were elevated following 4OI treatment (Figures 6C-6F and 7C-7F). *In vitro* study further revealed that 4OI at a superphysiologic level directly induced cell death and the release of DAMPs (HMGB1 and SQSTM1) in peritoneal macrophages (Figures 6I-6K). Overall, these studies indicate that STING1-mediated itaconate production promotes, rather than inhibits, the development of sepsis.

## Discussion

TLRs are evolutionally conserved pattern recognition receptors that detect specific PAMPs to active innate immune responses (Akira and Takeda, 2004). In this study, we found a regulatory mechanism for ACOD1 expression by coupling TLR and STING1 signals (Figure 7I). The activation of STING1 alone by ligands or a gain-of-function mutation is not sufficient to trigger ACOD1 expression. However, activated STING1 leads to increased sensitivity and response to inducible ACOD1 expression and itaconate production following stimulation with several TLR ligands, including LPS. Our findings not only provide insights into the regulation mechanism of immunometabolism (Jung et al., 2019; O'Neill et al., 2016), but also challenge current views on the anti-inflammatory activity of itaconate *in vivo* (Liao et al., 2019; Mills et al., 2018).

Although ACOD1 was originally described as an LPS-inducing gene in 1995 (Lee et al., 1995), the key function of ACOD1 in mediating itaconate production has not been studied until recently (Wu et al., 2020). ACOD1 is a mitochondrial protein, mainly expressed in myeloid cells, and its inducible expression can be used as a marker and regulator of inflammation during various infections (Wu et al., 2020). We proved that the STING1-MYD88 complex is a signal hub that mediates inducible ACOD1 expression in response to ligands of TLR1, TRL2, TRL4, TRL5, or TLR6. The function of STING1 in mediating LPS-induced ACOD1 expression depends on MYD88, rather than CGAS, which indicates that the STING1 signal pathway contributes to gene expression under different stimulations.

Signal transduction is a complex process that depends on stimuli and environment. Although CGAS was originally found to be required for STING1 activation during viral infection, recent studies have also reported a CGAS-independent STING1 pathway in response to different stimuli, including viral infection (Holm et al., 2016; Suschak et al., 2016; Unterholzner and Dunphy, 2019). Our current study suggests that MYD88 plays an alternative role in mediating STING1 activity in response to TLR ligands. cGAMP or other ligands of STING may also be produced in a CGAS-independent manner (Carozza et al., 2020; Wan et al., 2020). In fact, *Sting*^*−/−*^ and *Cgas*^*−/−*^ mice have overlapping and distinct phenotypes in disease models of infection and immunity (Suschak et al., 2016; Yum et al., 2021). An increasing number of natural or synthetic STING1 ligands have been discovered. It is still not excluded that CGAS may be involved in STING1-dependent ACOD1 expression under certain conditions, especially in pathological DNA damage situations (Motwani et al., 2019).

We provide experimental evidence that STING1 forms a protein complex with MYD88, which is necessary for the inducible expression of ACOD1. These findings may also establish a model to explain the interaction of STING1 and TLR signaling in the production of pro-inflammatory cytokines during infection (Tesser et al., 2021; Zeng et al., 2017). Although previous studies have shown that ACOD1 is implicated in the antiviral response (Daniels et al., 2019; Ren et al., 2016), we did not observe that TLR3, TLR7, TLR8, and TLR9 ligands induce ACOD1 expression in human monocytes. Thus, the production of ACOD1 in virus infection may not come directly from nucleic acid ligands. In contrast, viral infection-related cytokines, such as IFNs, likely are a stimulator of ACOD1 upregulation (Degrandi et al., 2009). Regardless, the STING1-MYD88 complex plays a major role in mediating ACOD1 expression during bacterial infection, especially in response to TLR1/2/4/5/6 signals.

The degradation of STING1 protein is a posttranslational modification mechanism that can inhibit an excessive innate immunity response (Pokatayev et al., 2020; Prabakaran et al., 2018). Our current study highlights the mechanism by which the formation of the STING1-MYD88 complex prevents STING1 degradation through an autophagic pathway, instead of the ubiquitin-proteasome system. Consequently, inhibiting the autophagic degradation of STING1 increases the level of STING1, providing a priming signal for subsequent MYD88-mediated ACOD1 expression. As STING1 also promotes autophagy (Gui et al., 2019; Zhang et al., 2021), it may establish negative feedback to control the expression and activation of STING1 during infection (Zhang et al., 2022b).

Using the 3′3'-cGAMP/LPS stimulation model, we further investigated the downstream transcription factors responsible for ACOD1 expression. Our data suggest that the transcription factor IRF3 coupled with JUN contributes to 3′3′-cGAMP/LPS-induced ACOD1 expression. In contrast, NF-κB (a well-known pro-inflammatory transcription factor in TLR signaling) is not required for this process. It is important to further identify the nuclear cofactors that facilitate the activation of IRF3 and JUN in controlling 3′3′-cGAMP/LPS-induced ACOD1 expression. Under different immune signal stimulation, the expression of inducible ACOD1 may depend on different transcription factors (Wu et al., 2020).

Our animal study raised a concern about the application of 4OI in infectious diseases. An initial study showed that 4OI has a mild protective effect on LPS-induced lethality in mice (Mills et al., 2018). The anti-inflammatory activity of itaconate or 4OI involves multiple mechanisms, such as the blocking of succinate dehydrogenase activity to reduce succinate-mediated inflammatory processes (Lampropoulou et al., 2016), upregulation of activating transcription factor 3 (ATF3) expression to limit IκBζ-mediated pro-inflammatory cytokine production (Bambouskova et al., 2018), or increasing nuclear factor erythroid 2-like 2 (NFE2L2) protein stability to induce anti-inflammatory gene expression (Mills et al., 2018). However, our sepsis mouse model did not find the protective activity of 4OI in a lethal infection. One possible explanation for this discrepancy could be owing to different infection models and timing of 4OI administration. Of note, 4OI treatment reversed the protection against septic death experienced by *Sting1*^*Mye−/−*^ mice. Although the mechanism is not clear, we demonstrated that itaconate causes cell death and DAMP (HMGB1 and SQSTM1) release, which is consistent with recent studies on the cytotoxicity of itaconate on cancer cells (Belosludtsev et al., 2020; Qu et al., 2021). HMGB1 and SQSTM1 are potential therapeutic targets for infection as well as sterile inflammation caused by tissue damage (Kang et al., 2014; Wang et al., 1999; Zhou et al., 2020; Zou et al., 2020).

In summary, the activation of the STING1 pathway in monocytes and macrophages can synergize with the MYD88 pathway to drive LPS-induced ACOD1 expression and itaconate production, which favors the development of septic death by the release of DAMPs. This innate immunity pathway may enhance our understanding of the immunopathological mechanisms of lethal infections.

### Limitations of the study

A limitation of our work is the use of cell lines rather than primary cells to study the relationship between MYD88 and STING1 in innate immunity. We also cannot rule out whether the MYD88-STING1 pathway is required for ACOD1-related inflammatory responses in other infectious diseases or tissue damage.

## STAR★Methods

### Key resources table

**Table:** 

REAGENT or RESOURCE	SOURCE	IDENTIFIER
**Antibodies**		

ACOD1	Cell Signaling Technology	77510; RRID:AB_2799901
ACOD1	Cell Signaling Technology	17805; RRID:N/A
STING1	Cell Signaling Technology	50494; RRID:AB_2799375
STING1	Abcam	ab288157; PRID:N/A
MYD88	Cell Signaling Technology	4283; RRID:AB_10547882
MYD88	Abcam	ab28763; RRID:AB_2146743
ATG5	Cell Signaling Technology	2630; RRID:AB_2062340
MAP1LC3B	Cell Signaling Technology	3868; RRID:AB_2137707
IRF3	Cell Signaling Technology	4302; RRID:AB_1904036
Phospho-IRF-3 (Ser396)	Cell Signaling Technology	4947; RRID:AB_823547
c-JUN	Cell Signaling Technology	9165; RRID:AB_2130165
Phospho-*c*-JUN (S63)	Cell Signaling Technology	3270; RRID:AB_2129575
NF-κB p65	Cell Signaling Technology	8242; RRID:AB_10859369
Phospho-NF-κB p65 (S536)	Cell Signaling Technology	3033; RRID:AB_331284
ACTB	Cell Signaling Technology	3700; RRID:AB_2242334
HRP-linked anti-mouse IgG secondary antibody	Cell Signaling Technology	7076; RRID:AB_330924
HRP-linked anti-rabbit IgG secondary antibody	Cell Signaling Technology	7074; RRID:AB_2099233

**Chemicals, peptides, and recombinant proteins**		

LPS O111:B4	Sigma-Aldrich	L2360
4-Octyl itaconate	MedChemExpress	HY-112675
Human TLR1 to TLR9 agonist	InvivoGen	tlrl-kit1hw
G3-YSD	InvivoGen	tlrl-ydna
G3-YSD control	InvivoGen	tlrl-ydnac
LyoVec	InvivoGen	lyec-12
3′3′-cGAMP	InvivoGen	tlrl-nacga
2′2′-cGAMP	InvivoGen	tlrl-nacga22
2′3′-cGAMP	InvivoGen	tlrl-nacga23-02
c-di-AMP	InvivoGen	tlrl-nacda
c-di-GMP	InvivoGen	tlrl-nacdg
Lipofectamine 3000	Invitrogen	L3000-015
Puromycin	InvivoGen	ant-pr-1
MG132	Selleck Chemicals	S2619
Chloroquine	Selleck Chemicals	S6999
Cell lysis buffer (10X)	Cell Signaling Technology	9803
SuperSignal West Pico Chemiluminescent Substrate	Thermo Fisher Scientific	34580
SuperSignal West Femto Maximum Sensitivity Substrate	Thermo Fisher Scientific	34095
QUANTI-Blue Solution	InvivoGen	rep-qbs1
QUANTI-Luc	InvivoGen	rep-qlc1
Hoechst 33258	Thermo Fisher Scientific	H3569

**Critical commercial assays**		

SQSTM1 ELISA kit	Enzo	ADI-900-212
HMGB1 ELISA kit	Shino-Test Corporation	326054329
D-dimer ELISA kit	MyBioSource	MBS723281
iScript cDNA Synthesis Kit	Bio-Rad	1708890
BCA Protein Assay Kit	Thermo Fisher Scientific	23225
Protein A Magnetic Beads	Millipore	LSKMAGA10
Proteome Profiler Human Phospho-Kinase Array	R&D Systems	ARY003B
SimpleChIP Enzymatic Chromatin IP Kit	Cell Signaling Technology	9003
QIAquick PCR Purification Kit	QIAGEN	28104
Cell Counting Kit-8 solutions	Bimake	B34304

**Experimental models: cell lines**		

Wild-type THP1	ATCC	TIB-202
THP1-Dual cells	InvivoGen	thpd-nfis
*STING1*^*−/−*^ THP1	InvivoGen	thpd-kostg
*IRF3*^*−/−*^ THP1	InvivoGen	thpd-koirf3
*STING1-V155M* THP1	InvivoGen	thpd-m155
*CGAS*^*−/−*^ THP1	InvivoGen	thpd-kocgas
*MYD88*^*−/−*^ THP1	InvivoGen	thpd-komyd
Wild-type RAW264.7	ATCC	TIB-71
*Irf3*^*−/−*^ RAW264.7	InvivoGen	rawl-koirf3

**Experimental models: organisms/strains**		

*Sting1*^*flox/flox*^ mice	The Jackson Laboratory	031670
*Lyz2/LysM-Cre* mice	The Jackson Laboratory	004781
C57BL/6 WT mice	The Jackson Laboratory	000664

**Oligonucleotides**		

Human *MYD88* shRNA	Sigma-Aldrich	TRCN0000008025
Human *ATG5* shRNA	Sigma-Aldrich	TRCN0000151963
Human *JUN* shRNA	Sigma-Aldrich	TRCN0000039589
Control shRNA	Sigma-Aldrich	SHC016V
Human *ACOD1* primers: 5′-TTCCAT GAATGCCAGATCAA-3′ and 5′-GGT TTTCTCCAGTGCCCATA-3′	Sigma-Aldrich	N/A
Mouse *Acod1* primers: 5′-GGTATCAT TCGGAGGAGCAAGAG-3′ and 5′-AC AGTGCTGGAGGTGTTGGAAC-3′	Sigma-Aldrich	N/A
Human *TNF* primers: 5′-CTCTTCTGCC TGCTGCACTTTG-3′ and 5′-TGGGCT ACAGGCTTGTCACTC-3′	Sigma-Aldrich	N/A
Human *IFNA1* primers: 5′-AGAAGG CTCCAGCCATCTCTGT-3′ and 5′-T GCTGGTAGAGTTCGGTGCAGA-3′	Sigma-Aldrich	N/A
Human *IL6* primers: 5′-AGACAGCC ACTCACCTCTTCAG-3′ and 5′-TTC TGCCAGTGCCTCTTTGCTG-3′	Sigma-Aldrich	N/A
Mouse *Rn18s RNA* primers: 5′-GCAA TTATTCCCCATGAACG-3′ and 5′-GG CCTCACTAAACCATCCAA-3′	Sigma-Aldrich	N/A
Human *RNA18S RNA* primers: 5′-CTACC ACATCCAAGGAAGCA-3′ and 5′-TTTTTC GTCACTACCTCCCCG-3′	Sigma-Aldrich	N/A
*STING1* cDNA	OriGene Technologies	SC321845

**Software and algorithms**		

Image Lab software version 6.1	Bio-Rad	http://www.bio-rad.com/en-us/product/image-lab-software?ID=KRE6P5E8Z
CFX Manager software version 3.1	Bio-Rad	http://www.bio-rad.com/en-us/sku/1845000-cfx-manager-software?ID=1845000
GraphPad Prism 8	GraphPad Software	https://www.graphpad.com/scientific-software/prism/
Quick Spots Image Analysis Software	Western Vision Software	http://www.wvision.com/QuickSpots.html

### Resource availability

#### Lead contact

Further information should be directed to and will be fulfilled by the lead contact Dr. Daolin Tang (daolin.tang@utsouthwestern.edu).

#### Materials availability

This study did not generate new unique reagents.

### Experimental model and subject details

#### Cell culture

Wild-type THP1 (TIB-202; female) and RAW264.7 (TIB-71; female) cell lines were obtained from the American Type Culture Collection (ATCC). The *STING1*^*−/−*^ (thpd-kostg), *IRF3*^*−/−*^ (thpd-koirf3; rawl-koirf3), *STING1-V155M* (thpd-m155), *CGAS*^*−/−*^ (thpd-kocgas), and *MYD88*^*−/−*^ (thpd-komyd) THP1 or RAW264.7 cell lines were obtained from InvivoGen. These cells were cultured in Dulbecco’s Modified Eagle’s Medium (DMEM; 11995073, Thermo Fisher Scientific) or RPMI 1640 (11875119, Thermo Fisher Scientific) supplemented with 10% heat-inactivated fetal bovine serum (TMS-013-B, Millipore) and 1% penicillin and streptomycin (15070-063, Thermo Fisher Scientific) at 37°C, 95% humidity, and 5% CO_2_. All cells used were authenticated using STR profiling, and mycoplasma testing was negative.

#### Animal models and treatment

C57BL/6J WT (000664), *Sting1*^*flox/flox*^ (031670), and *Lyz2*^*Cre*^ (004781) mice were obtained from the Jackson Laboratory. Myeloid cell-specific *Sting1*-deficient mice (*Sting1*^*Mye−/−*^) were generated by crossing the *Sting1*^*flox/flox*^ mice with *Lyz2*^*Cre*^ mice. Mice were housed with their littermates in groups of 4 or 5 animals per cage and kept on a regular 12-h light and dark cycle (7:00-19:00 light period). Food and water were available *ad libitum*. Experiments were carried out under pathogen-free conditions with randomly chosen littermates of the same sex (female or male [1:1]), matched by age (8–10 weeks old) and body weight (22–26 g weight). The health status of mouse lines was routinely checked by veterinary staff. We conducted all animal care and experimentation in accordance with the Association for Assessment and Accreditation of Laboratory Animal Care guidelines (http://www.aaalac.org) and with approval from institutional animal care and use committees.

##### Poly-microbial sepsis model

Sepsis was induced in male or female C57BL/6J mice (8–10 weeks old, 22 to 26 g weight, female or male [1:1]) using a surgical procedure termed CLP (Chen et al., 2019; Deng et al., 2018; Kang et al., 2016, 2018; Zeng et al., 2017; Zhang et al., 2020). Briefly, anesthesia was induced with ketamine (80–100 mg/kg/i.p.) and xylazine (10–12.5 mg/kg/i.p.). A small midline abdominal incision was made and the cecum was exteriorized and ligated with 4-0 silk immediately distal to the ileocecal valve without causing intestinal obstruction. The cecum was then punctured once with a 22-gauge needle. The abdomen was closed in two layers and mice were injected subcutaneously with 1 mL Ringer’s solution, including analgesia (0.05 mg/kg buprenorphine). Then 4OI (50 mg/kg) or vehicle were repeatedly administered intraperitoneally to mice at 2, 24, 48, and 72 h after CLP.

##### Endotoxemia model

LPS (*E*. *coli 0111*:*B4*, L4391, Sigma-Aldrich) was dissolved in PBS. Male or female C57BL/6J mice (8–10 weeks old, 22 to 26 g weight, female or male [1:1]) were intraperitoneally administered a single dose of LPS (15 mg/kg). Then 4OI (50 mg/kg) or vehicle were repeatedly administered intraperitoneally to mice at 2, 24, 48, and 72 h after LPS.

Survival was observed for up to 10 days. Blood was collected from anesthetized mice by cardiac puncture using heparinized syringes. Plasma was further obtained from anticoagulated whole blood after removing the blood cells by a centrifugation (2000 g × 15 min) at 4°C.

### Method details

#### ELISA and itaconate analysis

Commercially available ELISA kits were used to measure the concentrations of D-dimer (MBS723281, MyBioSource), HMGB1 (ST51011, IBL International), and SQSTM1 (ADI-900-212, Enzo) in the indicated samples. Measurement of ALT and BUN in the plasma was performed using a Catalyst Dx Chemistry Analyzer (IDEXX). Intracellular itaconate concentration was assayed by a liquid chromatography-tandem mass spectrometry (LC-MS/MS) method (Tan et al., 2020). In short, to minimize the chance of cell metabolite degradation, cells were lysed by adding ice-cold 80% methanol/water (v/v). The internal standard solution (^13^C_5_-itaconate; sc-495554, Santa Cruz) was also prepared in 80% methanol/water. Samples and standards were analyzed using a Triple Quad 5500 LC-MS/MS system (SCIEX).

#### RNAi and gene transfection

All pre-designed shRNA constructs in a lentiviral format were purchased from Sigma-Aldrich, as described in Key resources table. We seeded 1 × 10^5^ cells in each well of a 12-well plate in 500 μL of complete medium and transduced them by lentiviral vectors at an MOI of 10:1. Transduction was carried out in the presence of polybrene (8 μg/mL; TR-1003-G, Sigma-Aldrich). After recovering with complete culture medium, puromycin (5 μg/mL; ant-pr-1, InvivoGen) was used for the selection of transduced cells. STING1 expression plasmid (SC321845, OriGene Technologies) was transfected into THP1 cells (1 × 10^6^) using Lipofectamine 3000 reagent (L3000-015, Invitrogen) according to manufacturer’s instruction. LyoVec (lyec-12, InvivoGen) was used as a nucleic acid complexing agent to facilitate the cellular entry of RNA or DNA-based oligonucleotides.

#### qPCR

Total RNA was extracted using an RNeasy Plus Kit (74134, QIAGEN) according to the manufacturer’s instructions. First-strand cDNA was synthesized from 1 μg of RNA using the iScript cDNA Synthesis kit (1708890, Bio-Rad). Briefly, 20 μL reactions were prepared by combining 4 μL of iScript Select reaction mix, 2 μL of gene-specific enhancer solution, 1 μL of reverse transcriptase, 1 μL of gene-specific assay pool (20×, 2 μM), and 12 μL of RNA diluted in RNase-free water. Quantitative real-time PCR was carried out using synthesized cDNA, with primers described in Key resources table, and SsoFast EvaGreen Supermix (172-5204, Bio-Rad). The data were normalized to *Rna18s* and the fold change was calculated via the 2^−ΔΔCt^ method (Deng et al., 2018). The relative concentrations of mRNA were expressed in arbitrary units based on the untreated group, which was assigned a value of 1.

#### Western blot analysis

Cells were lysed in Cell Lysis Buffer (9803, Cell Signaling Technology) with protease inhibitor cocktail (G6521, Promega) and phosphatase inhibitor cocktail (P0044, Sigma-Aldrich) (Chen et al., 2022; Li et al., 2021b; Liu et al., 2022). Cleared lysates were resolved by SDS-PAGE (3450124, Bio-Rad) and then transferred onto PVDF membranes (1704273, Bio-Rad). The membranes were blocked with Tris-buffered saline Tween 20 (TBST; 9997, Cell Signaling Technology) containing 5% nonfat dry milk (9999, Cell Signaling Technology) for 1 h at room temperature and then incubated with the indicated primary antibodies (1:1000) overnight at 4°C. After being washed with TBST, the membranes were incubated with an HRP-linked anti-mouse IgG secondary antibody (1:1000; 7076, Cell Signaling Technology) or HRP-linked anti-rabbit IgG secondary antibody (1:1000; 7074, Cell Signaling Technology) for 1 h at room temperature. The membranes were washed three times in TBST and then visualized and analyzed with a ChemiDoc Touch Imaging System (1708370, Bio-Rad).

#### Immunoprecipitation analysis

Cells were lysed at 4°C in ice-cold RIPA buffer (9806, Cell Signaling Technology) with protease inhibitor cocktail (G6521, Promega), and cell lysates were cleared by a brief centrifugation (12000*g*, 10 min) (Tang et al., 2010). Concentrations of proteins in the supernatant were determined by a BCA Protein Assay Kit (7780, Cell Signaling Technology). Prior to immunoprecipitation, samples containing equal amounts of proteins were pre-cleared with protein A agarose beads (9863, Cell Signaling Technology) at 4°C for 3 h, and subsequently incubated with various irrelevant IgG or specific antibodies (3–5 μg/mL) in the presence of protein A agarose beads for 2 h or overnight at 4°C with gentle shaking (Li et al., 2021c; Zhu et al., 2017). Following incubation, protein A agarose beads were washed extensively with phosphate-buffered saline and proteins were eluted by boiling in 2 × sodium dodecyl sulfate sample buffer (LC2676, Thermo Fisher Scientific) before electrophoresis of the sodium dodecyl sulfate polyacrylamide gel.

#### ChIP

ChIP was performed using a SimpleChIP Enzymatic Chromatin IP Kit (9003, Cell Signaling Technology) according to manufacturer’s instructions (Deng et al., 2018). Briefly, THP1 cells (1 × 10^7^) were cross-linked with 1% fresh formaldehyde and incubated for 10 min at room temperature. Cells were lysed and chromatin was digested to obtain DNA fragments from 150 to 900 bp. Chromatin fragments were immunoprecipitated with anti-JUN or anti-IRF3 antibodies (1:50) at 4°C overnight with rotation and then incubated with protein G magnetic beads at 4°C for 2 h. After eluting chromatin from antibodies and reversing formaldehyde-induced cross-linking, the DNA was purified using a QIAquick PCR Purification Kit (28104, QIAGEN). One-20th of the immunoprecipitated DNA was used in qPCR. Results were shown as a percentage of input.

#### Proteome profiler antibody array analysis

We used a Human Phospho-Kinase Array Kit (ARY003B, R&D Systems), which has membrane-based sandwich immunoassays, to assay the effects of 3′3′-cGAMP (10 μg/mL) and LPS (500 ng/mL) on kinase activation at 6 h. Captured antibodies spotted in duplicate on nitrocellulose membranes bond to specific target proteins present in the sample (step 1). Captured proteins were detected with biotinylated detection antibodies (step 2) and then visualized using chemiluminescent detection reagents (step 3). The signal produced was proportional to the amount of analyte bound. The intensities of bands were analyzed with Quick Spots Image Analysis Software (Zeng et al., 2017).

#### Transcription factor activity assay

THP1-Dual cells (InvivoGen) express a Lucia luciferase gene under the transcription control of IFN and a secreted embryonic alkaline phosphatase (SEAP) reporter gene under the transcription control of NF-κB. They were used to simultaneously measure the activity of NF-κB and IRF pathways. Indicated THP1-Dual cells (WT, *STING1*^*−/−*^, and *MYD88*^*−/−*^) in a 96-well plate (1 × 10^5^/well) were treated with 3′3′-cGAMP (10 μg/mL) and LPS (500 ng/mL) for 6 h. The cell culture supernatant was collected and then the activity of NF-κB and IRF were measured using QUANTI-Blue (a SEAP detection reagent) and QUANTI-Luc (a luciferase detection reagent), respectively.

#### Cytotoxicity assays

Cells were seeded at 5 × 10^4^ cells per well into 96-well plates and incubated with the indicated treatments. Subsequently, 100 μL of fresh medium was added to cells containing 10 μL of Cell Counting Kit-8 solutions (B34304, Bimake) and incubated for 1.5 h in 5% CO_2_ at 37°C. Absorbance at 450 nm was measured using a microplate reader.

#### Immunofluorescence analysis

Cells were cultured on glass coverslips and fixed in 3% formaldehyde for 30 min at room temperature prior to detergent extraction with 0.1% Triton X-100 for 10 min at 25°C (Dai et al., 2020; Li et al., 2021a, 2021c). Coverslips were saturated with 2% bovine serum albumin (BSA) in phosphate-buffered saline (PBS) for 1 h at room temperature and processed for immunofluorescence with primary antibodies, followed by Alexa Fluor 488- or Cy3-conjugated secondary antibodies. Nuclear morphology was analyzed with the fluorescent dye Hoechst 33258. Images were taken with a ZEISS LSM 800 confocal microscope (ZEISS, Germany).

### Quantification and statistical analysis

GraphPad Prism 8.4.3 was used to collect and analyze data. Unpaired Student’s *t* tests were used to compare the means of two groups. A one-way or two-way analysis of variance (ANOVA) with Tukey’s multiple comparisons test was used for comparisons among the different groups. Log-rank tests were used to compare differences in mortality rates between groups. A p value of <0.05 was considered statistically significant. The exact value of n within the figures is indicated in the figure legends. We did not exclude samples or animals. No statistical methods were used to predetermine sample sizes, but our sample sizes are similar to those generally employed in the field.

## Data Availability

Data: The authors declare that all data supporting the findings of this study are available within the article or available from the corresponding author upon reasonable request. Code: This study did not generate any code. Other items: Any additional information required to reanalyze the data reported in this paper is available from the lead contact upon request.
